# Reproductive performance of grazing dairy cows genetically divergent in genetic merit for feed intake

**DOI:** 10.3168/jdsc.2025-0955

**Published:** 2026-05-22

**Authors:** M. Williams, D.P. Berry

**Affiliations:** Animal and Grassland Research and Innovation Centre, Teagasc, Moorepark, Fermoy, Co. Cork, Ireland P61 C996

## Abstract

Feed-related costs represent one of the largest variable costs in dairy herds, and, consequently, many countries now include explicit measures of feed intake or feed efficiency in their dairy total merit indexes. Nevertheless, there is a paucity of research investigating the effects on reproductive performance from genetic divergence on feed intake and feed efficiency within the framework of a total merit index. Given that the repercussions of compromised reproductive performance in pasture-based dairy systems are severe, the objective of the present study was to characterize the reproductive performance of grazing dairy cows stratified on their EBV for energy intake. After edits, 14,451 net energy intake (NEI) records from 5,060 lactations on 2,521 cows were available from 5 separate Irish research farms; reproductive records were available for 2,508 of these cows across 9,788 lactations. The cows were a mixture of Holstein-Friesian, Jersey, Montbéliarde, Norwegian Red, and their crosses. Estimated breeding values for NEI and NEI adjusted for energy sinks (NEI_adj_) were derived using phenotypic data from 2 reference datasets based on a selection of years or a selection of farms; this approach was chosen to reflect practical genetic evaluation scenarios where EBVs are estimated from historical data or data from a limited number of farms but applied to animals born in the future or in different herds. Cows in the test datasets based on year of calving or herd were individually stratified, within experimental treatment by year and season of calving, into 4 strata based on their within-breed EBV for NEI, as well as separately for NEI_adj_. Subsequently, predicted marginal means for calving interval, interval from calving to first service, and number of services were estimated within each EBV stratum of the test datasets using linear mixed models; logistic regression models were used to generate predicted probability estimates for a series of pregnancy and submission binary traits. In both test dataset scenarios (by year of recording and by farm), differences between NEI and NEI_adj_ EBV strata were observed for calving interval, interval from calving to first service, 21-d submission rate, and 21-d calving rate. After correcting for multiple testing, however, differences among the strata remained for only 1 of the submission traits; in the test dataset of farm, cows with higher NEI_adj_ EBVs were more likely to be served within 21 d of the breeding season, provided they had calved at least 30 d earlier. The findings of the present study suggest that, within the genetic variability present in the studied population, selection for NEI or NEI_adj_ would, in the short term at least, likely have minimal influence on the reproductive performance of grazing dairy cows, with the possible exception of submission rate.

## Highlights

• Cows genetically divergent for feed intake have similar reproductive performance.
• Including feed intake in a total merit index will not influence fertility in the short term.
• These findings will help inform the potential of direct selection for grass feed intake.

## Graphical Abstract

Many countries now include measures of feed intake or feed efficiency in their dairy breeding objectives. Nevertheless, there is a paucity of research investigating the effects of genetic selection for feed intake and feed efficiency on reproductive performance. The findings of this study indicate that, among cows already selected under a total merit index, ranking them based on genetic merit for net energy intake resulted in minimal differences in reproductive performance between cows with high and low genetic merit for net energy intake. The findings of the present study suggest that selecting for net energy intake, in the short term, at least, as part of a total merit index, would likely have minimal influence on the fertility performance of grazing dairy cows; this information could help inform the effects of direct selection for feed intake among grazing dairy cows.

**Figure:**
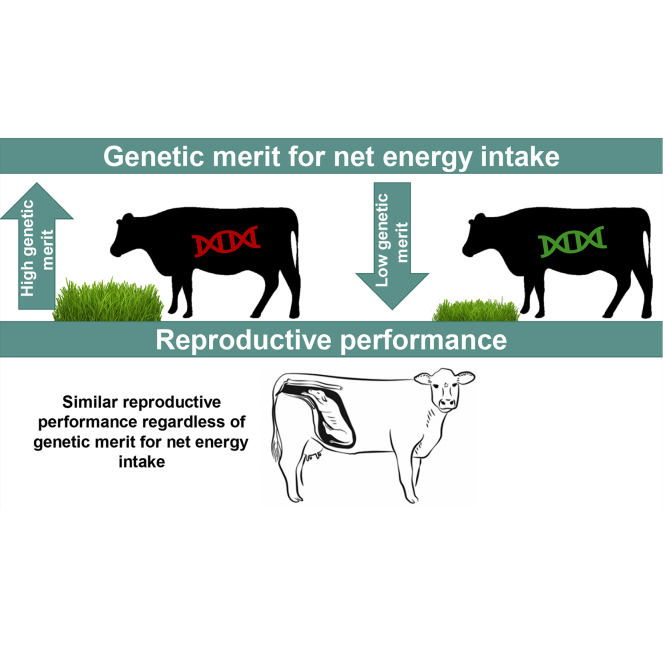

Profitable pasture-based dairy production relies on synchronizing peak milk production with peak pasture availability; this means that cows must conceive and calve within a narrow season. Hence, poor reproductive performance is more deleterious in pasture-based production systems than in year-round confinement production systems; delayed or failed conception in pasture-based production systems likely leads to the cow being culled prematurely.

Feed-related costs represent ~44% of total production costs on Irish dairy farms, which are predominantly pasture-based (Teagasc, 2024). Consequently, many dairy cow total merit indexes, including those in Australia (Pryce et al., 2015; Byrne et al., 2016), the Netherlands (CRV, 2022), the United Kingdom (AHDB, 2025), and the United States (CDCB, 2021), now include explicit measures of feed intake or feed efficiency. Several studies have recently validated the usefulness of breeding values for feed intake or feed efficiency (or both) to change the respective phenotypic measures (Davis et al., 2014; Pryce et al., 2014; Williams et al., 2026). Nevertheless, relatively few studies have investigated the relationship between genetic selection for feed efficiency and reproductive performance in dairy cows. Vallimont et al. (2013) reported unfavorable genetic correlations between feed efficiency and conception rate in a study of 970 US dairy cows in tiestall barns. Pryce et al. (2014) documented only weak phenotypic associations between residual feed intake and reproductive performance in 157 cows ranked on their residual feed intake as growing animals. Hence, careful consideration is needed before feed intake or feed efficiency traits are widely included in selection indices, particularly in pasture-based dairy production systems. Therefore, the objective of the present study was to characterize the reproductive performance of grazing dairy cows stratified on their EBV for energy intake or energy efficiency.

Records of individual cow daily pasture DMI estimates and concentrate DMI, collected between the years 1998 and 2022, inclusive, were available from the Animal and Grassland Research and Innovation Centre, Teagasc Moorepark, Co. Cork, Ireland. Data originated from 5 different research farms. Dry matter intake was calculated as pasture DMI plus concentrate DMI. Details of the animal management, the n-alkane procedure to estimate DMI at pasture, and pasture digestibility metrics have been described in detail by Williams et al. (2026); the n-alkane technique to estimate individual cow feed intake was repeated, on average, 2.7 times per lactation and, on average, 5.6 times per cow. Dry matter intake records from cows without a known sire were removed, as were DMI records more than 3 SD from the parity by stage of lactation mean. Body condition score was recorded every ~3 wk on a scale from 1 to 5 (increments of 0.25), and BW was recorded every 1 to 3 wk using an electronic scale. After all edits, 14,451 DMI records from 5,060 lactations on 2,521 cows were available. The dataset comprised 10,543 records from Holstein-Friesian cows (73%), 422 records from Jersey cows (2.9%), 88 records from Montbéliarde cows (0.6%), 40 records from Norwegian Red cows (0.3%), and 3,357 records from crossbred cows (23%); breed proportions were based on the recorded pedigree.

Calving dates and service dates were available for cows with DMI records. After the removal of obvious data errors (i.e., cows calving multiple times in short intervals or cows being served before calving), calving dates and services dates were available from 10,878 and 8,648 lactations, respectively. Calving season per year was defined separately for primiparous and multiparous cows; all animals within the herd were used when defining calving season whether the animals had a DMI record or not. The start date of the calving season was defined as the first calving date within a herd-year, where at least 5 cows calved within the subsequent 14 d. The start of the breeding season was defined within each herd-year as the first date, between March and October, when 5 multiparous cows were serviced within the subsequent 14 d; all animals within the herd were used when defining the start of the breeding season, irrespective of whether or not they had a DMI record. Where 2 service records for the same cow were within 5 d of each other, only the latter record was retained. Number of services was defined as the number of services a cow received in a lactation after removal of services that were fewer than 5 d apart.

Interval from calving to first service was defined as the number of days between calving and the first recorded service postpartum. Calving interval was defined as the number of days between consecutive calvings; only calving intervals >300 d were retained. Where a calving-to-first-service interval record <150 d existed, then calving interval records up to 800 d were retained; otherwise a calving interval upper threshold of 600 d was used.

Binary reproductive traits were defined to capture calving, submission, and pregnancy outcomes. Calving in the first 21 d (**CALV21**) and 42 d (**CALV42**) of the calving season were defined as whether a cow calved within these respective time periods (1) or not (0); cows calving within 14 d before the start of the calving season were considered as calving within both windows, to account for premature births and short gestations. Submission rate traits included whether a cow was serviced in the first 21 d of the breeding season (**SR21**), irrespective of calving date, and a restricted definition (**SR21b**) for cows that calved at least 30 d before the start of the breeding season; cows without a recorded service were set to missing for both traits. Pregnancy traits included pregnant within the first 42 d of the breeding season (**PR42**), defined based on a subsequent calving consistent with a gestation length of 265 to 300 d, and pregnant to first service (**PRFS**), where cows were classified as pregnant (1) or not (0), with cows receiving more than 1 service classified as not pregnant to first service; pregnant to first service was confirmed where a subsequent calving date record was available that could confirm pregnancy using the same gestation length definition used for PR42. Pregnancy outcomes were inferred from subsequent calving records, as information on abortion events was not available.

Only calving records from cows in parity ≤10 were retained, and parity was categorized as 1, 2, 3, 4, or ≥5. Heterosis and recombination loss coefficients were calculated for each cow as follows:

$$\mathrm{heterosis}=1-\sum_{i=1}^{n}\mathrm{sir}e_{i}\times \mathrm{da}m_{i};$$

$$\mathrm{recombination}=1-\sum_{i=1}^{n}\frac{{\mathrm{sire}}_{i}^{2}+{\mathrm{dam}}_{i}^{2}}{2},$$

where sire*_i_* and dam*_i_* are the proportion of breed *i* in the sire and dam, respectively. Heterosis was stratified into 12 classes (0%, >0% and ≤10%, >10% and ≤20%, >20% and ≤30%, >30% and ≤40%, >40% and ≤50%, >50% and ≤60%, >60% and ≤70%, >70% and ≤80%, >80% and ≤90%, >90% and ≤99%, and >99%). Recombination loss was stratified into 7 classes (0%, >0% and ≤10%, >10% and ≤20%, >20% and ≤30%, >30% and ≤40%, >40% and ≤50%, and >50%). Contemporary group was defined as experimental group by year and season of calving (January to June, inclusive, and July to December, inclusive), and contemporary groups with <10 calving records were removed. After all edits, 9,788 calving records from 2,508 cows remained; of these records, 7,946 had a corresponding calving interval and 7,927 had a corresponding interval from calving to first service and number of services. Of the 5,060 cow-years with DMI records, 4,799 had calving records, of which 4,118 also had corresponding service records.

Before the estimation of the variance components for net energy intake (**NEI**), the available DMI records were divided into a reference dataset and a corresponding test dataset; DMI phenotypes in the test dataset were masked. Two approaches to defining the reference populations used to estimate the EBVs were investigated: (1) by year of recording, or (2) by farm. In the by-year scenario, the test dataset comprised all DMI records collected between the years 2019 and 2022, inclusive, with the corresponding reference dataset consisting of all DMI records collected before 2019. The DMI phenotypes in the test dataset were masked and not used in the estimation of EBVs; EBVs for these animals were predicted solely based on their genetic relationships with animals in the reference dataset. In the by-farm scenario, DMI data from 1 farm served as the test dataset, with the data from the other 4 farms serving as the reference dataset; this was iterated 5 times so that every farm was the test farm once. All DMI records from animals in the test population were excluded from the reference in both test scenarios. The number of NEI phenotypes in each of the by-farm reference and test datasets are described elsewhere (Williams et al., 2026).

The variance components for NEI were estimated separately for each reference dataset using univariate animal linear mixed models in ASReml (Gilmour et al., 2008) with parity, DIM class (and its interaction with parity), heterosis class, recombination class, and contemporary group as fixed effects; additive genetic and permanent environmental effects (within and across lactations) were included as random effects. The pedigree of all animals was traced to founders and assigned to genetic groups by breed; the pedigree included 30,213 animals, with up to 23 generations traced. In a separate analysis, the variance components for NEI adjusted for energy sinks (**NEI_adj_**) were estimated, using the same model but with NEL, metabolic live weight, live weight change, BCS, and BCS change also included as covariates in the model. As described in Williams et al. (2026), EBV were generated for both NEI and NEI_adj_ (NEI adjusted for NEL, metabolic live weight, live weight change, BCS, and BCS change) using the NEI phenotypes from each reference dataset in the Mix99 software suite (Lidauer et al., 2017). Breed proportions for all of the main dairy breeds (Holstein, Friesian, Jersey, Ayrshire, Brown Swiss, Montbéliarde, Norwegian Red, and Normande) were fitted as linear covariates in the genetic evaluations, such that the resulting animal solutions were within-breed EBVs. Within-breed EBVs for the test animals were estimated through their expected relationships, via the numerator relationship matrix, with the phenotyped animals in the reference dataset.

To avoid confounding EBV strata with breed composition, each lactation within test population was stratified, within experimental treatment by year and season of calving contemporary group, into 4 strata based on the within-breed EBV of the cow for NEI and NEI_adj_. Although feed intake records were restricted to data collected since 2019 for the by-year test dataset, calving and reproductive records from earlier years were retained for all cows, provided there were at least 10 records within the contemporary group. In the by-year test population, the number of lactations for each of the NEI strata varied from 587 to 657. In the by-farm test dataset, the number of lactations per NEI stratum varied from 2,360 to 2,753. Separate logistic mixed models in PROC GLIMMIX in SAS 9.4 (SAS Institute Inc.) were used to estimate the predicted probability of a positive outcome for each binary trait (PR42, PRFS, CALV21, CALV42, SR21, and SR21b) within each NEI EBV and NEI_adj_ EBV stratum. Predicted marginal means for the continuous calving and reproductive traits (calving-to-first-service interval, calving interval, and number of services) were estimated for each EBV stratum using linear mixed models in PROC MIXED in SAS 9.4; pairwise comparisons among EBV strata were adjusted for multiple testing using the Tukey–Kramer method. The fixed effects of parity (1, 2, 3, 4, ≥5), heterosis class, recombination class, and EBV stratum were included in all models. Experimental treatment by year and season of calving contemporary group nested with farm was included as a random effect in all models, as was a repeated cow effect. The repeated cow effect was not included when estimating the predicted marginal means for calving interval in the by-year test dataset due to the limited number of cows with repeated records. Each farm was sequentially treated as a test dataset, with each lactation in a single farm assigned to 1 of the 4 NEI EBV strata within that farm. The datasets of all 5 farms were combined for subsequent analysis of the association between NEI EBV stratum and performance; the justification for combining the farm test datasets was to increase the statistical power for detecting differences among strata. In a separate series of analyses, the reproductive performance traits were regressed on the EBV for NEI or NEI_adj_ treated as a continuous variable; this was undertaken using the same linear or logistic mixed models as previously described but where EBV stratum was substituted with the respective covariate.

The predicted marginal means for the continuous reproductive traits, along with the predicted probability of a positive outcome for each binary trait, by EBV stratum for NEI and NEI_adj_, are shown in Table 1, Table 2, respectively. In the by-year test dataset, cows stratified on their EBV for NEI differed only in interval from calving to first service and CALV21 (Table 1), whereas, when stratified by EBV for NEI_adj_, differences existed only for calving-to-first-service interval and SR21 (Table 2). In the by-farm test dataset, cows stratified on EBV for NEI differed only in calving interval (Table 1), whereas, when stratified on EBV for NEI_adj_, differences were observed only for SR21b (Table 2). Nevertheless, the trend in mean reproductive performance across EBV strata was not consistent either across traits or test datasets, indicating that no clear differences in reproductive performance were detected within the limits of this study. Additionally, when the EBV for NEI or NEI_adj_ were fitted as continuous variables in place of EBV stratum, these analyses yielded consistent results, with no associations observed with any of the continuous or binary traits, except for 2 cases: in the farm test dataset, NEI EBV was associated with PR42 (regression coefficient = −0.14, SE = 0.07), and NEI_adj_ EBV was associated with SR21b (regression coefficient = −0.10, SE = 0.04).

**Table 1:** Mean EBVs for net energy intake (NEI EBV; SE) and predicted marginal means (SE) for calving interval, interval from calving to first service, and number of services, along with the mean predicted probability (SEM) of achieving SR21, SR21b, PR42, PRFS, CALV42, and CAL21 in by-year or by-farm test datasets

Trait and test set^1^	NEI EBV stratum 1	NEI EBV stratum 2	NEI EBV stratum 3	NEI EBV stratum 4	*P*-value
By year					
NEI EBV (UFL)	2.28 (0.01)^a^	2.39 (0.01)^b^	2.53 (0.01)^c^	2.76 (0.01)^d^	<0.001
Calving interval (d)	375.3 (3.2)	379.0 (3.17)	375.0 (3.0)	374.2 (3.1)	0.3109
Calving to first service (d)	80.2 (2.1)^ab^	81.6 (2.1)^a^	78.7 (2.0)^b^	78.2 (2.0)^b^	0.0601
Number of services	1.52 (0.05)	1.50 (0.05)	1.60 (0.04)	1.54 (0.05)	0.2116
SR21	0.87 (0.02)	0.86 (0.02)	0.89 (0.02)	0.87 (0.02)	0.4099
SR21b	0.80 (0.03)	0.79 (0.03)	0.81 (0.03)	0.79 (0.03)	0.6345
CALV21	0.30 (0.03)^ab^	0.32 (0.03)^a^	0.26 (0.03)^b^	0.27 (0.03)^ab^	0.0752
CALV42	0.68 (0.03)	0.69 (0.03)	0.65 (0.03)	0.67 (0.03)	0.5387
PRFS	0.58 (0.04)	0.58 (0.04)	0.54 (0.03)	0.58 (0.03)	0.3633
PR42	0.82 (0.03)	0.80 (0.03)	0.83 (0.03)	0.81 (0.03)	0.6915
By farm					
NEI EBV (UFL)	2.14 (0.01)^a^	2.35 (0.01)^b^	2.55 (0.01)^c^	2.79 (0.01)^d^	<0.001
Calving interval (d)	381.8 (2.0)^a^	385.8 (2.0)^ab^	382.2 (1.9)^ab^	386.1 (2.0)^b^	0.0508
Calving to first service (d)	78.6 (0.8)	77.9 (0.8)	77.6 (0.8)	77.7 (0.8)	0.4830
Number of services	1.66 (0.03)	1.68 (0.03)	1.68 (0.02)	1.66 (0.03)	0.8759
SR21	0.86 (0.01)	0.86 (0.01)	0.87 (0.01)	0.86 (0.01)	0.6587
SR21b	0.78 (0.02)	0.78 (0.02)	0.79 (0.01)	0.79 (0.02)	0.3928
CALV21	0.27 (0.02)	0.28 (0.02)	0.27 (0.02)	0.26 (0.02)	0.5241
CALV42	0.70 (0.02)	0.68 (0.02)	0.68 (0.02)	0.68 (0.02)	0.4899
PRFS	0.54 (0.01)	0.54 (0.01)	0.52 (0.01)	0.55 (0.01)	0.4543
PR42	0.71 (0.02)	0.67 (0.02)	0.68 (0.02)	0.69 (0.02)	0.1108

a–d Means within a row with different superscripts differ significantly (*P* < 0.05).

1 NEI = net energy intake; SR21 = serviced in the first 21 d of the breeding season; SR21b = serviced in the first 21 d of the breeding season and calved ≥30 d before the breeding season; CALV21 = calving in the first 21 d of the calving season; CALV42 = calving in the first 42 d of the calving season; PRFS = pregnancy to first service; PR42 = pregnant within the first 42 d of the breeding season.

**Table 2:** Mean EBVs for net energy intake adjusted energy sinks (NEI_adj_ EBV; SE) and predicted marginal means (SE) for calving interval, interval from calving to first service, and number of services, and the mean predicted probability (SEM) of achieving SR21, SR21b, CALV42, and CAL21 and PR42, PRFS in by-year or by-farm test datasets

Trait and test set^1^	NEI_adj_ EBV stratum 1	NEI_adj_ EBV stratum 2	NEI_adj_ EBV stratum 3	NEI_adj_ EBV stratum 4	*P*-value
By year					
NEI_adj_ EBV (UFL)	−8.92 (0.01)^a^	−8.77 (0.01)^b^	−8.76 (0.01)^b^	−8.64 (0.01)^c^	<0.001
Calving interval (d)	374.2 (3.2)	377.4 (3.1)	375.0 (3.0)	375.9 (3.2)	0.6936
Calving to first service (d)	78.0 (2.1)^a^	81.2 (2.0)^b^	78.5 (2.0)^a^	80.3 (2.1)^ab^	0.0710
Number of services	1.62 (0.05)	1.49 (0.05)	1.52 (0.05)	1.55 (0.05)	0.1006
SR21	0.89 (0.02)^a^	0.84 (0.03)^b^	0.89 (0.02)^a^	0.87 (0.02)^ab^	0.0278
SR21b	0.80 (0.03)	0.79 (0.03)	0.81 (0.03)	0.79 (0.03)	0.7037
CALV21	0.28 (0.03)	0.30 (0.03)	0.28 (0.03)	0.28 (0.03)	0.8904
CALV42	0.64 (0.04)	0.68 (0.03)	0.68 (0.03)	0.65 (0.03)	0.4466
PRFS	0.52 (0.04)	0.59 (0.03)	0.59 (0.03)	0.56 (0.04)	0.1172
PR42	0.81 (0.03)	0.83 (0.03)	0.81 (0.03)	0.82 (0.03)	0.7924
By farm					
NEI_adj_ EBV (UFL)	−8.75 (0.02)^a^	−8.54 (0.01)^b^	−8.41 (0.01)^c^	−8.23 (0.02)^d^	<0.001
Calving interval (d)	382.6 (2.1)	384.2 (2.0)	382.7 (1.9)	386.5 (2.0)	0.2069
Calving to first service (d)	78.0 (0.8)	78.3 (0.8)	77.4 (0.8)	78.0 (0.8)	0.6396
Number of services	1.67 (0.03)	1.65 (0.03)	1.68 (0.03)	1.67 (0.03)	0.8274
SR21	0.86 (0.01)	0.85 (0.01)	0.87 (0.01)	0.86 (0.01)	0.3192
SR21b	0.78 (0.02)^ab^	0.77 (0.02)^a^	0.79 (0.01)^ab^	0.80 (0.01)^b^	0.0499
CALV21	0.27 (0.01)	0.27 (0.02)	0.26 (0.01)	0.27 (0.02)	0.7241
CALV42	0.69 (0.02)	0.68 (0.02)	0.68 (0.02)	0.70 (0.02)	0.5432
PRFS	0.53 (0.01)	0.54 (0.01)	0.53 (0.01)	0.54 (0.01)	0.7498
PR42	0.72 (0.02)	0.69 (0.02)	0.71 (0.02)	0.72 (0.02)	0.1373

a–d Means within a row with different superscripts differ significantly (*P* < 0.05).

1 NEI_adj_ = net energy intake adjusted for NEL, metabolic live weight, live weight change, BCS, and BCS change; SR21 = serviced in the first 21 d of the breeding season; SR21b = serviced in the first 21 d of the breeding season and calved ≥30 d before the breeding season; CALV21 = calving in the first 21 d of the calving season; CALV42 = calving in the first 42 d of the calving season; PRFS = pregnancy to first service; PR42 = pregnant within the first 42 d of the breeding season.

When adjusted for multiple testing, no difference in reproductive performance was found among any EBV strata in either test dataset, except for SR21b across different NEI_adj_ strata. When limited to cows that calved at least 30 d before the start of the breeding season (i.e., SR21b) in the by-farm test, cows ranked in the bottom 25% on genetic merit for NEI_adj_ had a lower submission rate in the first 21 d of the breeding season than cows with genetic merit for higher NEI_adj_. The within-breed genetic SD of NEI and NE_Iadj_ estimated from the cows in the present study were 0.78 and 0.50, respectively (Williams et al., 2026). This means that the mean difference in EBV between the extreme strata was less than 1 genetic SD for both traits in both test datasets. Therefore, it is possible that if a more genetically divergent group of animals were compared, differences in reproductive performance might have been more apparent. Having said that, the experimental populations contributing to this dataset were designed to maximize genetic variability; nevertheless, because EBVs are subject to shrinkage toward the population mean, the observable differences between animals are reduced, which likely contributed to the relatively small differences between strata. Nonetheless, using the same dataset of cows used in the present study, Williams et al. (2026) reported that cows of genetically higher NEI produced more milk, were heavier, and produced more milk relative to their metabolic BW than contemporaries who genetically were expected to consume less, with the trends in these traits being relatively consistent across NEI strata.

The Irish total merit index, the Economic Breeding Index (EBI; Berry et al., 2007) combines milk production, reproductive performance, and other economically important traits to promote balanced genetic improvement; a particularly strong emphasis is placed on reproductive performance and survival, given its importance in pasture-based dairy systems. As interest grows in incorporating feed intake and efficiency traits, it is crucial to ensure that selection for improved (daily) efficiency does not compromise reproductive performance or other traits that underpin herd-level efficiency. Estimated breeding values for NEI and NEI_adj_ used in the present study have previously been shown to predict phenotypic differences in feed intake and related performance traits in independent datasets, with differences of up to 1.0 unité fourragère du lait (UFL) per day between extreme EBV strata (Williams et al., 2026). Given, therefore, the importance of reproductive performance, and the relatively small number of animals per stratum in the present study, a potential risk of Type II errors exists, especially for the binary traits. To explore this, cows were stratified again into 4 strata but on their national genetic evaluation parental average EBV for calving interval; the mean difference in calving-interval EBV between cows in the extreme strata was 7.8 d when tested by year and 8.8 d when tested by farm, equating to 1.4 to 1.6 of the genetic SD used in the national genetic evaluation. Cows in the extreme quartile groups for calving interval EBV differed (*P* < 0.05) for several reproductive traits, including calving interval, interval from calving to first service, number of services, and measures of submission and pregnancy rates. These differences were observed in both the by-year and by-farm test populations; nevertheless, the trends across strata were not consistent for all traits or test datasets. Nonetheless, cows in the best stratum for EBV for calving interval also had better mean reproductive performance than contemporaries in the worst calving-interval EBV stratum. The presence of differences in reproductive traits when cows of varying genetic merit for calving interval were compared confirms that sufficient statistical power existed to detect differences if they truly existed. Additional variance–covariance analyses between NEI or NEI_adj_ and reproductive performance yielded genetic correlations close to zero; for example, the genetic correlation between NEI and calving interval and NEI_adj_ and calving interval were 0.014 (SE = 0.035) and 0.002 (SE = 0.046), respectively, supporting the absence of a meaningful genetic association between energy intake and reproductive performance.

The lack of consistent differences in reproductive performance among NEI or NEI_adj_ strata after correcting for multiple testing indicates only that no clear associations were detected within the limits of this study. This does not, however, imply that genetic selection solely for NEI will not affect reproductive performance, especially given that there was less than 1 genetic SD for NEI between the strata in the present study. Rather, it simply implies that genetic selection for differences in NEI in the short term is unlikely to influence reproductive success.

In conclusion, the limited differences in reproductive performance among cows varying in genetic merit for energy intake suggest that selection, in the short term, at least, for either higher or lower energy intake, within the framework of a total merit index that also includes reproductive performance, could contribute to a change in daily feed intake with minimal effect on reproductive performance. Nonetheless, these results only apply in grazing dairy systems where NEI was estimated using the n-alkane technique.
